# Suicidal and Infanticidal Mania

**Published:** 1829-04-01

**Authors:** 


**HITCHIN DISPENSARY.**

Case of Suicidal and Infanticidal Mania. Under the care of Dr. Hawkins.

E. B. a young, and hitherto healthy, woman, the mother of two children, in humble life, but not in indigence, applied at the Hitchin Dispensary, in consequence of the most miserable feelings of gloom and despondency, accompanied by a strong, and by her own account, an almost irresistible propensity, or temptation, as she termed it, to destroy her infant. This feeling first came upon her about a week before, when the child was a month old; and she was now sunk into an extreme state of dejection. She begged to be continually watched, lest she should yield to this strange propensity. The appetite is bad—bowels loose—stools dark and offensive—has occasionally discharged portions of tape worm from the bowels. Pulse natural; sleeps ill. This account is taken from the Dispensary report-book, October, 1824, and the treatment need not be mentioned, as the symptoms continued without alteration till March, 1825, when the patient took the small-pox. During the eruption, the mind was serene and happy, and she was free from the dreadful temptation by which she had been previously harrassed; but, upon the subsidence of the small-pox, the disease returned with its former horrors.

About the middle of April, the disease, without any apparent cause, began to decline, and she was, at the end of the month discharged from the dispensary by her own request: her child was now six months old. She nursed it herself from its birth, and continued to do so till it was twelve months old. She remained free from any disorder until the Spring of this year, 1828, when she had another child, and, about a month after the birth of it, she was assailed by this propensity to destroy it. The symptoms continued till the child was half a year old; and from that time have gradually declined. Occasionally for a few days, a sort of metastasis takes place; the propensity to destroy the infant entirely subsides, and the place of it is supplied by an equally strong disposition to suicide. It is worthy of remark, that during the most distressing periods of her disease, she is perfectly aware of the atrocity of the deed she is so powerfully impelled to, and prays fervently to be enabled to withstand so great a temptation. She has repeatedly told Dr. Hawkins, that the inclination to destroy her child has been so powerful, that she should certainly have yielded to it if she had suffered herself to use a knife even at her meals;—for a knife is the instrument which she feels necessitated to employ in the perpetration of the act. Whilst this extraordinary state lasts, the bowels are uniformly relaxed, and the stools of a dark colour and offensive odour. She has suckled all her children for twelve months: she has had three children, but this dreadful state of mind has supervened on the birth of the two last only. It may be proper to observe that, when suffering from any bodily indisposition, the mind is serene and happy, and free from any kind of morbid feeling. This poor woman is by no means deficient in affection for her infant even in the most trying period of her disease. This case appears of much importance in a medico-legal consideration. Is she of sound or unsound mind? And supposing, in this case, suicide were committed, and a verdict of insanity were returned, should not the same verdict be returned supposing infanticide were the crime which the jury had to consider? The case altogether presents a very remarkable moral phenomenon.

